# From Genotype to Cardiac Phenotype: Cardiovascular Involvement in Syndromic and Metabolic Disorders

**DOI:** 10.3390/ijms27167080

**Published:** 2026-08-07

**Authors:** Chung-Lin Lee, Ya-Hui Chang, Chih-Kuang Chuang, Huei-Ching Chiu, Yuan-Rong Tu, Yun-Ting Lo, Jun-Yi Wu, Hsiang-Yu Lin, Shuan-Pei Lin

**Affiliations:** 1 Department of Pediatrics, MacKay Memorial Hospital, No. 92, Sec. 2, Zhongshan N. Rd., Taipei 104217, Taiwan; clampcage@gmail.com (C.-L.L.); wish1001026@gmail.com (Y.-H.C.); g880a01@mmh.org.tw (H.-C.C.); 2 Institute of Clinical Medicine, National Yang-Ming Chiao-Tung University, Taipei 112304, Taiwan; 3 International Rare Disease Centre, MacKay Memorial Hospital, Taipei 104217, Taiwan; andy11tw.e347@mmh.org.tw (Y.-T.L.); wl01723138@gmail.com (J.-Y.W.); 4 Department of Medicine, Mackay Medical College, New Taipei City 252005, Taiwan; 5 Department of Nursing, Mackay Junior College of Medicine, Nursing and Management, New Taipei City 252005, Taiwan; 6 Division of Genetics and Metabolism, Department of Medical Research, MacKay Memorial Hospital, Taipei 104217, Taiwan; mmhcck@gmail.com (C.-K.C.); likemaruko@hotmail.com (Y.-R.T.); 7 College of Medicine, Fu-Jen Catholic University, New Taipei City 242062, Taiwan; 8 Department of Medical Research, China Medical University Hospital, Taichung 404327, Taiwan; 9 Department of Infant and Child Care, National Taipei University of Nursing and Health Sciences, Taipei 112303, Taiwan

**Keywords:** next-generation sequencing, genotype–phenotype correlation, echocardiography, mucopolysaccharidosis, RASopathy, cardiomyopathy, congenital heart disease, inborn errors of metabolism, pediatric genetics

## Abstract

Cardiovascular disease is a leading cause of morbidity and premature mortality in many inherited syndromic and metabolic disorders. However, its cardiac manifestations are often recognized late and are rarely described collectively within a single cohort. We reviewed eight years of outsourced next-generation sequencing (NGS) requested through the pediatric genetics service of a single tertiary center in Taiwan and identified 22 patients with molecularly confirmed genetic disorders and documented cardiovascular involvement. For each patient, the causative genotype—including lysosomal storage diseases, RASopathies, CHARGE syndrome, connective-tissue disorders, primary cardiomyopathies and channelopathies, neuromuscular disorders, contiguous-gene syndromes, and other metabolic and syndromic conditions—was mapped to a structured echocardiographic phenotype. Septal defects or shunts and valvular regurgitation were the most common findings (10/22 and 9/22, respectively), followed by septal hypertrophy, valvular stenosis, and great-vessel or aortic abnormalities. Two children had left ventricular systolic dysfunction, and one died following an out-of-hospital cardiac arrest. Several cardiac lesions clustered by disease category, most notably valvular thickening in mucopolysaccharidoses and elastin arteriopathy in Williams–Beuren syndrome. These genotype-to-cardiac phenotype patterns support the need for gene-informed, systematic cardiac surveillance rather than symptom-driven referral in children with these disorders.

## 1. Introduction

Next-generation sequencing (NGS) has evolved from a research tool into a first-line diagnostic test for children with suspected genetic disorders. In large clinical series, exome or genome sequencing provides a molecular diagnosis for approximately one-third of patients referred with heterogeneous or nonspecific clinical presentations [1,2]. A recent meta-analysis of more than 24,000 pediatric probands reported a pooled diagnostic yield of approximately 34% for genome-wide sequencing [3], while clinical exome panels have shown comparable performance, even in resource-limited settings [4]. Because a single test can now interrogate hundreds to thousands of genes simultaneously, its diagnostic utility extends across the full spectrum of Mendelian disorders, from inborn errors of metabolism to multisystem malformation syndromes [5,6,7].

The heart is one of the organs most frequently affected within this expanding phenotypic spectrum, and cardiovascular disease is a major contributor to morbidity and premature mortality in many of these conditions. Progressive valvular thickening and regurgitation are common in mucopolysaccharidoses and continue to progress with age despite enzyme replacement therapy [8]. Cardiomyopathy is a frequent and sometimes fatal complication of organic acidemias [9]. Pulmonary stenosis and septal defects are associated with specific RASopathy genotypes [10]. In inherited cardiomyopathies and channelopathies, the causative variant increasingly guides arrhythmic risk assessment and decisions regarding implantable defibrillator therapy [11]. Nevertheless, these cardiac manifestations are often recognized late because clinical attention at diagnosis is understandably focused on the presenting neurological, skeletal, or dysmorphic features.

The cardiac features of these disorders, although clinically diverse, arise through a comparatively small number of distinct molecular mechanisms, and grouping the conditions by mechanism rather than by clinical label brings their cardiovascular biology into sharper focus. In the lysosomal storage diseases, deficient substrate catabolism leads to progressive accumulation of glycosaminoglycans in valvular and vascular tissue and to a characteristic infiltrative valvulopathy [8]. In the RASopathies, constitutive activation of RAS–MAPK signalling promotes cardiomyocyte hypertrophy and abnormal semilunar-valve formation [10]. Disruption of the sarcomere and of its cytoskeletal and desmosomal attachments produces the hypertrophic, dilated, and arrhythmogenic cardiomyopathies, in which the genotype increasingly guides arrhythmic-risk stratification [11]. Defects in extracellular-matrix proteins such as elastin and fibrillar collagen weaken the great vessels and cardiac valves, whereas dysregulation of developmental programmes—chromatin remodelling and Notch and mTOR signalling—underlies the structural malformations, arterial stenoses, and cardiac tumours of the malformation syndromes. Seen in this light, the heart is a shared downstream target of otherwise unrelated genes, and the causative variant carries information not only about the diagnosis but about the specific cardiac lesion to anticipate.

This mechanistic perspective has practical consequences that current care does not fully exploit. Cardiac assessment in a child with a genetic diagnosis is still largely prompted by symptoms or found incidentally, and surveillance schedules, where they exist at all, are disease-specific and inconsistently applied. A view that spans disorders—linking each genotype to the pathway it disrupts and to the lesion that pathway produces—could support earlier, gene-informed cardiac evaluation and, increasingly, the selection of pathway-targeted therapies. Such an integrated genotype-to-phenotype picture is nonetheless rarely assembled because individual centres encounter these conditions one diagnosis at a time.

Most of the current knowledge regarding cardiovascular involvement in these disorders comes from single-disease cohorts, and these conditions are rarely examined together. Consequently, it remains difficult to distinguish cardiac lesions that are shared across different genetic disorders from those that are disease-specific, despite the importance of this information in guiding echocardiographic surveillance and follow-up. The situation is further complicated when more than one pathogenic variant is identified in the same patient, a scenario that NGS increasingly uncovers and that may obscure the relationship between genotype and cardiac phenotype [12,13,14].

To address this gap, we reviewed eight years of outsourced NGS requested through the pediatric genetics service of a single tertiary center and identified patients with molecularly confirmed genetic disorders and documented cardiovascular involvement. For each patient, the causative genotype was mapped to a structured echocardiographic phenotype to describe recurrent genotype-to-cardiac phenotype patterns and support the use of gene-informed, rather than symptom-driven, cardiac surveillance in this patient population.

## 2. Results

### 2.1. Patients and Molecular Diagnoses

**Figure 1:**
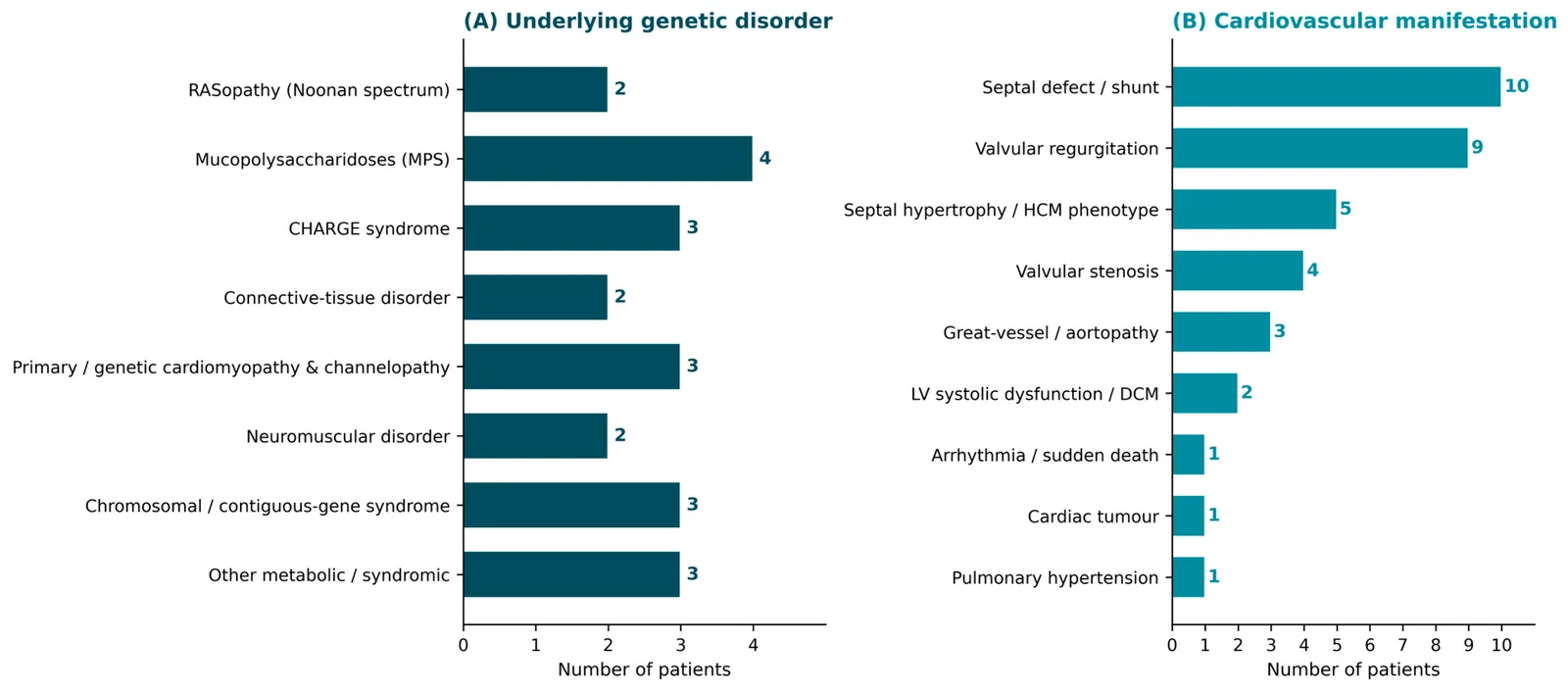
Spectrum of genetic disorders and cardiovascular manifestations in the 22-patient cohort. (**A**) Distribution of patients by underlying genetic disorder category. (**B**) Frequency of cardiovascular manifestations across the cohort. Because individual patients could have more than one type of lesion, the categories are not mutually exclusive, and the total count exceeds the number of patients. Abbreviations: DCM, dilated cardiomyopathy; HCM, hypertrophic cardiomyopathy; LV, left ventricular; MPS, mucopolysaccharidosis.

Twenty-two patients (11 females and 11 males) met the inclusion criteria of having a molecularly established genetic diagnosis and documented cardiovascular involvement. Their underlying disorders spanned eight categories (Figure 1A, Table 1): mucopolysaccharidoses constituted the largest group (*n* = 4), followed by CHARGE syndrome, primary cardiomyopathies and channelopathies, contiguous-gene syndromes, and other metabolic or syndromic conditions (*n* = 3 each), and RASopathies, connective-tissue disorders, and neuromuscular disorders (*n* = 2 each). Twenty distinct genes or loci were represented, ranging from single-gene defects, such as *CHD7*, *KMT2D*, *FLNC*, and *JAG1*, to contiguous-gene deletions at 22q11.2, 16p13.3, and 7q11.23. Case-level genotypes and echocardiographic findings are summarized in Table 1.

During the eight-year study period, 244 test requests were submitted for 242 patients through the division, of whom 94 received a molecularly established genetic diagnosis recorded in the divisional testing log; echocardiography had been performed in the majority of these patients, although cardiac assessment is not recorded systematically in the log and an exact count is therefore not recoverable. Thirty-one of these records documented cardiovascular findings and were screened in detail for the present analysis. Nine were excluded—one because the clinical records were no longer available, five because the molecular diagnosis could not be confirmed, two because neither cardiovascular involvement nor an echocardiogram was documented, and one because the cardiac finding could not be attributed to the genetic diagnosis—leaving the 22 patients reported here. The full selection process and all exclusion reasons are shown in Section 4.3 and detailed in Section 4.2.

### 2.2. Spectrum of Cardiovascular Involvement

Two lesion types predominated in the cohort (Figure 1B, Table 2): septal defects or shunts, which were present in 10 of 22 patients (45%), and valvular regurgitation, observed in nine patients (41%). Septal hypertrophy was present in five patients (23%) and valvular stenosis in four (18%), whereas great-vessel or aortic abnormalities were identified in three patients (14%). Left ventricular systolic dysfunction was observed in two patients (9%), while pulmonary hypertension, a cardiac tumor, and an arrhythmic sudden death event were each recorded in one patient. More than half of the cohort (13/22) had more than one type of cardiovascular lesion; therefore, these categories were not mutually exclusive. Left ventricular ejection fraction was preserved in most patients, except the two cases described below. Two children—one with MPS II and the other carrying a DSG2 cardiomyopathy-associated variant—had structurally normal baseline echocardiograms and were included based on their genotype-associated cardiac risk.

These proportions should be read with the design of the study in mind. The denominator is a small, highly selected group of 22 patients who already had both a confirmed molecular diagnosis and a cardiac assessment, so the percentages describe the internal composition of this cohort and are not estimates of the prevalence of any lesion in the underlying disorders. They are given alongside the absolute counts throughout for this reason.

Cardiac assessment was not confined to a single time point. The 21 patients with an echocardiogram had a median of two studies each (range 1–39), and the median interval between the first and the last study was 2.3 years (range 0–15.1); sixteen patients had at least two studies and seven had been followed for five years or more. Age at genetic diagnosis ranged from the neonatal period to 29.8 years and age at the most recent echocardiogram from 0 to 31.8 years. Per-patient ages, the number of studies, the duration of cardiac follow-up, and the individual quantitative measurements are given in Table S1.

Where serial studies were available, they showed how strongly the cardiac phenotype depended on age. The patient with the truncating FLNC variant had been imaged 39 times between the ages of 0.3 and 15.3 years: the ejection fraction was 59% in infancy and fluctuated between 50% and 65% through the first decade, fell below 40% from about the age of ten, and reached a nadir of 18% before implantation of a cardiac resynchronisation defibrillator, after which it recovered to between 30% and 37%. The patient with the PLEC-related myopathy followed a shorter but equally instructive course, with an ejection fraction of 63% at 17.4 years, 65% at 21.7 years, and 34% at 22.8 years. Neither trajectory would have been apparent from a single study, and both illustrate why a normal or near-normal early examination cannot be taken as reassurance in these genotypes.

### 2.3. Genotype-to-Cardiac-Phenotype Patterns

**Figure 2:**
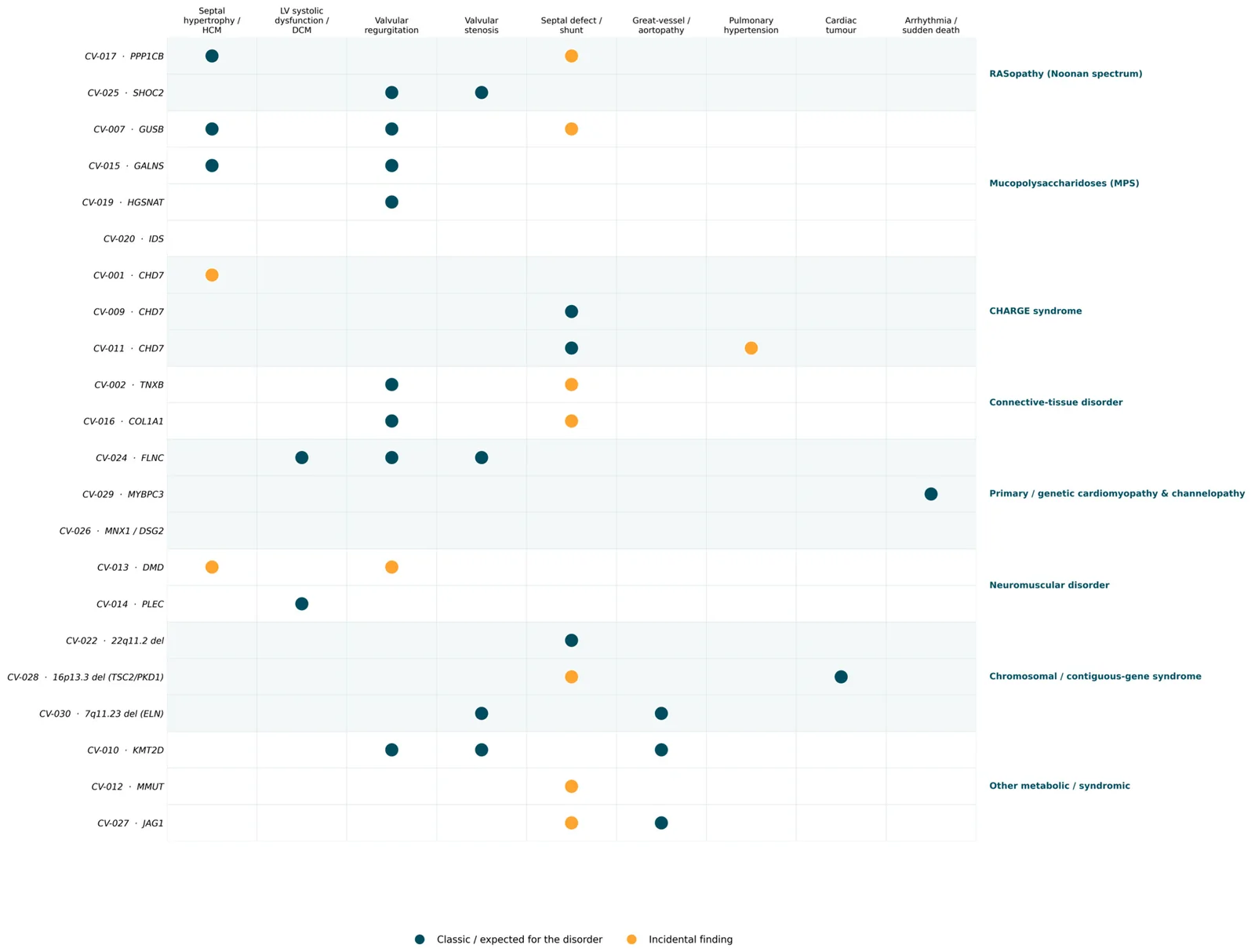
Genotype-to-cardiac-phenotype matrix for the 22 patients. Each row represents an individual patient, identified by study number and causative gene or cytogenetic locus and grouped by disorder category (shown on the right). Each column represents a category of cardiovascular lesion. Filled circles indicate the presence of a lesion: dark circles denote lesions that are classic or expected for the underlying disorder, whereas amber circles indicate incidental findings (see key). Patients with structurally normal baseline echocardiograms have no circles in any column. DCM, dilated cardiomyopathy; HCM, hypertrophic cardiomyopathy; LV, left ventricular.

When cardiovascular lesions were examined according to the underlying genotype (Figure 2), several recurring patterns emerged within disease categories. In the mucopolysaccharidoses, valve involvement with septal thickening was the predominant finding. Patients with MPS VII and MPS IVA showed mitral and aortic valve leaflet thickening with regurgitation and mild septal hypertrophy, whereas the patient with MPS IIIC had isolated mild aortic regurgitation. The two patients with RASopathies exhibited the expected right-sided and hypertrophic lesions, including pulmonary valve stenosis in the SHOC2 case and septal hypertrophy in the PPP1CB case. All three patients with *CHD7*-related CHARGE syndrome presented with ductal or atrial shunts, including patent ductus arteriosus treated either surgically or by transcatheter device closure and an atrial septal defect, consistent with the conotruncal tendency of this syndrome. The patients with connective-tissue disorders (TNXB and COL1A1) each had a small atrial septal defect accompanied by mild atrioventricular valve regurgitation.

The most severe cardiac phenotypes were observed in the primary cardiomyopathy and neuromuscular disorder groups. The child carrying a truncating FLNC variant had dilated cardiomyopathy with a left ventricular ejection fraction of 24–30%, a bicuspid aortic valve, and moderate mitral regurgitation. He had previously undergone ventricular septal occlusion and implantation of a cardiac resynchronization therapy defibrillator. A patient carrying an MYBPC3 variant of uncertain significance experienced an out-of-hospital cardiac arrest with suspected long QT syndrome or another channelopathy and subsequently progressed to brain death; no echocardiographic data were available. Among the neuromuscular disorders, a boy of four with Duchenne muscular dystrophy had preserved systolic function (ejection fraction 62%) but already showed mild septal hypertrophy together with trivial-to-mild tricuspid regurgitation, and left-ventricular hypertrophy was evident on his electrocardiogram. In contrast, a young man with PLEC-related myopathy developed interval left ventricular dilation and reduced fractional shortening between the 2024 and 2025 echocardiographic studies, indicating emerging cardiomyopathy.

The contiguous-gene syndromes demonstrated several disease-specific cardiovascular manifestations. The patient with tuberous sclerosis had multiple cardiac rhabdomyomas involving both ventricles and the right atrium. The patient with Williams–Beuren syndrome had supravalvular and peripheral pulmonary stenosis together with a small ascending aorta, consistent with the elastin arteriopathy characteristic of this deletion syndrome, whereas the patient with the 22q11.2 deletion had a spontaneously closed ventricular septal defect. Among the remaining disorders, the patient with Kabuki syndrome (*KMT2D*) had the most complex cardiovascular anatomy, including a persistent left superior vena cava, bicuspid aortic valve, aortic-arch anomaly, and mitral valve prolapse with regurgitation. The patient with Alagille syndrome (*JAG1*) exhibited peripheral pulmonary stenosis, a characteristic feature of the disorder. As illustrated in Figure 2, most cardiovascular lesions were classified as classic manifestations of their respective disorders, whereas only a few findings, such as the small atrial shunts observed in the connective-tissue disorder and organic acidemia cases, were considered incidental.

### 2.4. Molecular Spectrum and Variant Classification

The 22 patients carried variants in 20 distinct genes or cytogenetic loci (Table 3). At the sequence level the changes were heterogeneous: ten missense, five splice-site, four nonsense, and four frameshift variants, together with three contiguous-gene deletions (at 7q11.23, 16p13.3, and 22q11.2). The inheritance patterns spanned the expected modes—autosomal-dominant single-nucleotide or small-indel variants (most often heterozygous, several de novo), biallelic (compound-heterozygous or homozygous) variants in the recessive lysosomal and metabolic disorders, and hemizygous variants in the X-linked conditions (*IDS* in MPS II and *DMD*). Twenty of the 22 diagnoses rested on variants classified as pathogenic or likely pathogenic under ACMG/AMP criteria; the two exceptions were variants of uncertain significance in *MYBPC3* and *TNXB*, retained because the clinical picture—and, for *MYBPC3*, the fatal arrhythmic phenotype—was strongly supportive.

**Figure 3:**
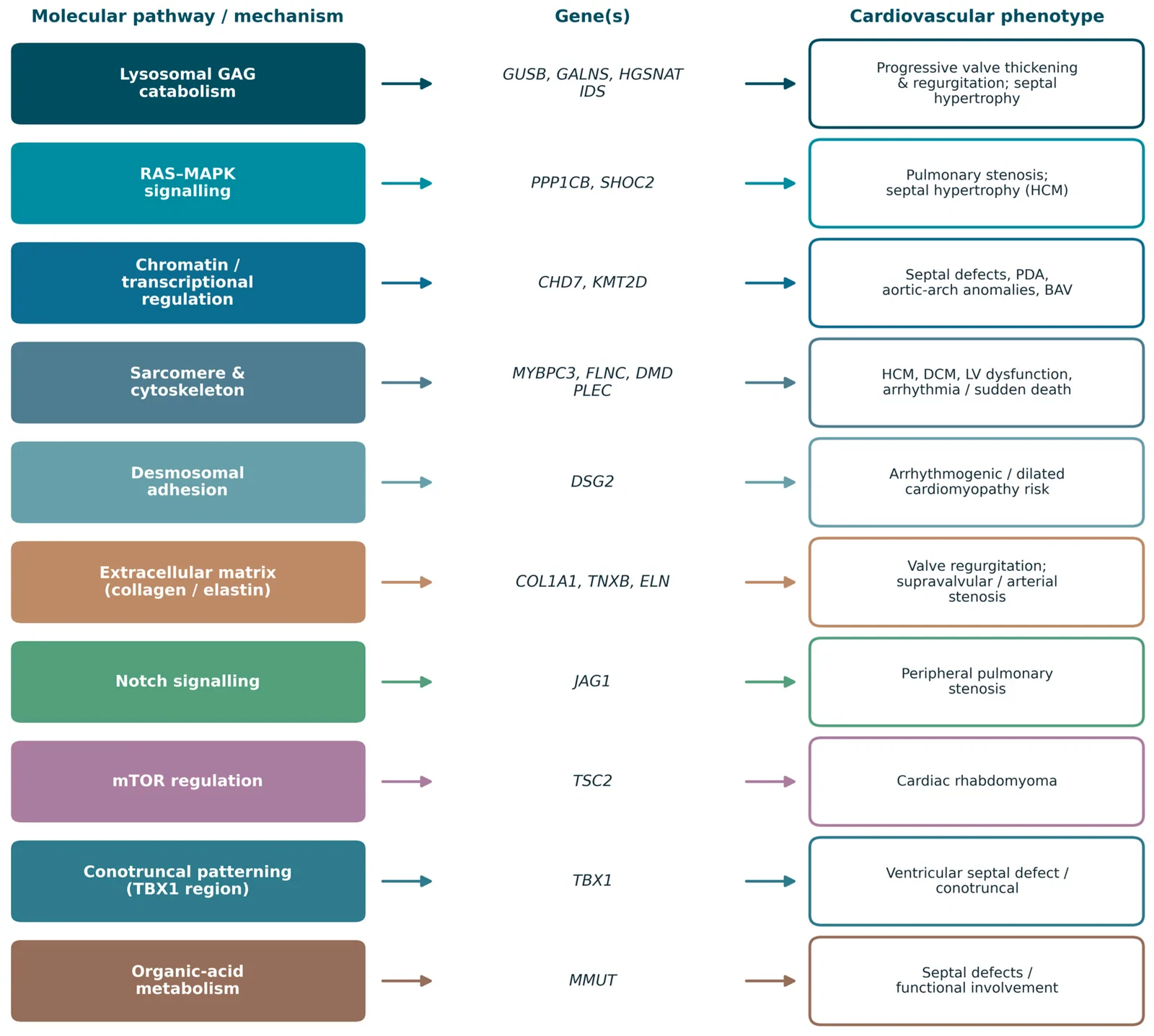
From molecular pathway to cardiac phenotype across the cohort. Each causative gene is grouped by the biological pathway it disrupts (left) and linked to the cardiovascular phenotype characteristic of that pathway (right). Abbreviations: BAV, bicuspid aortic valve; DCM, dilated cardiomyopathy; HCM, hypertrophic cardiomyopathy; LV, left ventricular; PDA, patent ductus arteriosus.

Grouping the genes by the pathway they disrupt made the genotype–phenotype structure of the cohort easier to see (Figure 3). The causative genes fell into ten functional categories: lysosomal glycosaminoglycan catabolism (*GUSB*, *GALNS*, *HGSNAT*, *IDS*); RAS–MAPK signalling (*PPP1CB*, *SHOC2*); chromatin and transcriptional regulation (*CHD7*, *KMT2D*); sarcomere and cytoskeletal integrity (*MYBPC3*, *FLNC*, *DMD*, *PLEC*); desmosomal adhesion (*DSG2*); extracellular-matrix and connective-tissue proteins (*COL1A1*, *TNXB*, *ELN*); Notch signalling (*JAG1*); mTOR regulation (*TSC2*); conotruncal patterning through the TBX1 region (22q11.2); and organic-acid metabolism (*MMUT*). The cardiac lesions tracked these categories closely—lysosomal genes with valve thickening and septal hypertrophy, RAS–MAPK genes with pulmonary stenosis and hypertrophy, sarcomeric and cytoskeletal genes with cardiomyopathy and arrhythmia, extracellular-matrix genes with valvar regurgitation and arterial stenosis, and the chromatin regulators with septal and outflow-tract defects—a correspondence developed further in Section 3.

## 3. Discussion

By assembling patients across the full range of disorders referred to a single genetics service, rather than focusing on one diagnosis at a time, we were able to examine how closely cardiovascular involvement follows the underlying genotype. In most of our 22 patients, the cardiac phenotype was consistent with that predicted by the molecular diagnosis: valve thickening with regurgitation in the mucopolysaccharidoses, pulmonary stenosis and septal hypertrophy in the RASopathies, ductal and atrial shunts in CHARGE syndrome, cardiac rhabdomyomas in tuberous sclerosis, and supravalvular and peripheral pulmonary stenosis—the hallmark elastin arteriopathy—in Williams–Beuren syndrome. Examining these disorders together helps distinguish lesions that recur across unrelated genotypes, chiefly septal defects and atrioventricular valve regurgitation, from those that are largely disease-specific and, in some cases, almost diagnostic.

These gene-linked patterns are consistent with previous reports from single-disease cohorts. The mucopolysaccharidosis-associated valvulopathy observed in our cohort—mitral and aortic valve thickening with regurgitation—mirrors the progressive glycosaminoglycan-driven valve disease described in longitudinal MPS cohorts, which progresses with age and is only partly modified by enzyme replacement therapy [8]. In RASopathies, the association between specific RAS/MAPK genotypes and pulmonary stenosis or hypertrophic changes is well established, with cardiac defects clustering around particular genes [10]. Likewise, cardiomyopathy is a recognized and sometimes fatal feature of organic acidemias, in which cardiac events contribute substantially to mortality [9]. Our findings provide a cross-disorder perspective that complements these previous observations.

At the severe end of the spectrum, the patient with FLNC-associated dilated cardiomyopathy in our cohort—a markedly reduced ejection fraction, a bicuspid aortic valve, and an implanted cardiac resynchronization therapy defibrillator—illustrates how a single truncating variant can lead to advanced heart failure and increased arrhythmic risk. Previous studies of FLNC variants have reported similar associations with dilated or arrhythmogenic cardiomyopathy, reduced ejection fraction, and sudden cardiac death [15]. The patient with an MYBPC3 variant who died following an out-of-hospital cardiac arrest highlights that the first cardiac manifestation of a cardiomyopathy- or channelopathy-associated gene may be a fatal arrhythmia. In both dilated and nondilated cardiomyopathies, genotype is increasingly used to stratify arrhythmic risk and guide decisions regarding implantable cardiac devices [11]. These cases support treating a high-risk cardiomyopathy genotype as clinically actionable.

The recurrent lesions can be traced to the specific molecular defect in each pathway. In the mucopolysaccharidoses, deficient lysosomal hydrolase activity (*GUSB*, *GALNS*, *HGSNAT*, *IDS*) causes progressive accumulation of glycosaminoglycans within valvular interstitial cells and the arterial wall; the resulting leaflet thickening, chordal infiltration, and increased ventricular mass generate the mitral and aortic regurgitation and septal hypertrophy seen here. Enzyme replacement improves systolic and diastolic myocardial indices and aortic stiffness but has little effect on established valve disease, which continues to progress even in treated patients [16]—an argument for lifelong echocardiographic follow-up irrespective of treatment status.

In the RASopathies, germline variants in *PPP1CB* and *SHOC2* converge on constitutive activation of the RAS–MAPK cascade, which drives cardiomyocyte hypertrophy and the abnormal semilunar-valve development that manifest as septal hypertrophy and pulmonary stenosis. This mechanistic link is now therapeutically actionable: the MEK inhibitor trametinib reduces contractile force and improves calcium handling in RASopathy hypertrophic myocardium ex vivo [17] and has produced measurable regression of outflow-tract obstruction and hypertrophy in severely affected infants [18], shifting these lesions from fixed structural problems toward pathway-targeted disease.

The cardiomyopathies in our cohort reflect disruption of the force-generating and force-transmitting apparatus of the cardiomyocyte at successive structural levels—the sarcomere (*MYBPC3*), the Z-disc and its filamin-C anchor (*FLNC*), the sarcolemmal dystrophin complex (*DMD*), the cytolinker plectin (*PLEC*), and the desmosome (*DSG2*). Truncating *FLNC* variants in particular cause haploinsufficiency of a Z-disc scaffolding protein and are strongly associated with dilated and arrhythmogenic phenotypes and sudden death [15], consistent with the severe *FLNC* case and the fatal *MYBPC3* event described above; because arrhythmia can precede or dominate the structural picture in this group, genotype is increasingly combined with imaging to stratify risk [11].

Two aspects of these cardiomyopathy genotypes deserve comment. First, the phenotypic range of *FLNC* extends beyond the dilated and arrhythmogenic forms: missense variants segregate with autosomal-dominant familial restrictive cardiomyopathy, in which affected individuals develop severe diastolic dysfunction and may require transplantation, and filamin-C aggregates are demonstrable in myocardial tissue [19]. Our patient carried a frameshift variant and presented with a dilated phenotype, but the wider filaminopathy spectrum is relevant when counselling families and when interpreting a truncating variant in an asymptomatic relative. Second, the *DSG2* variant in our cohort (c.81+1G>A) is heterozygous. This distinction matters, because hemizygous and homozygous loss-of-function *DSG2* genotypes—including a homozygous splice-site variant and a nonsense variant combined with a large deletion—have been shown to cause recessive arrhythmogenic cardiomyopathy with an early onset, sometimes in families without an apparent history [20]. A single heterozygous splice-site variant such as ours therefore confers risk that is neither negligible nor equivalent to a biallelic genotype, which is why we regarded this child as warranting longitudinal surveillance rather than as having established disease, and why segregation analysis and copy-number assessment of the second allele are worth pursuing.

A further mechanism—defective extracellular matrix—underlies the connective-tissue and elastin phenotypes. Haploinsufficiency of *ELN* within the 7q11.23 deletion reduces elastic-fibre content in the arterial wall, producing the progressive supravalvular and peripheral pulmonary stenosis and small ascending aorta characteristic of the elastin arteriopathy [21]; the same lesion in non-syndromic *ELN* loss shows that a single dosage-sensitive matrix gene is sufficient to generate it. Analogous defects in fibrillar collagen (*COL1A1*) and tenascin-X (*TNXB*) weaken valve and vessel architecture and account for the atrioventricular-valve regurgitation observed in the osteogenesis imperfecta and Ehlers–Danlos patients.

The structural congenital defects, by contrast, clustered in genes that regulate transcription and tissue patterning. *CHD7* and *KMT2D* encode chromatin-modifying enzymes that act within the TBX1 regulatory network governing second-heart-field and neural-crest development; damaging variants in such chromatin regulators are enriched among conotruncal and outflow-tract defects and appear to modify cardiac risk in 22q11.2 deletion as well [22], linking our CHARGE, Kabuki, and 22q11.2 patients through a shared developmental programme. Two further pathways complete the picture: reduced *JAG1*–NOTCH signalling impairs pulmonary-artery smooth-muscle development and produces the fibrous medial thickening that underlies the peripheral pulmonary stenosis of Alagille syndrome [23], and loss of *TSC2*-mediated restraint of mTOR drives the cardiac rhabdomyomas of tuberous sclerosis, which regress with the mTOR inhibitor everolimus [24].

Taken together, these pathways indicate that “cardiovascular involvement” in genetic disease is not a single entity but the read-out of distinct molecular lesions, several of which are now druggable—enzyme replacement and, increasingly, gene therapy in the mucopolysaccharidoses [25], MEK inhibition in the RASopathies [17,18], and mTOR inhibition in tuberous sclerosis [24]. This reinforces the value of pairing every molecular diagnosis with a structured cardiac phenotype, because the genotype increasingly determines not only which lesion to expect but also which treatment might modify it.

The practical implication is that cardiac surveillance in this population should be guided by genotype rather than initiated only after symptoms develop. Two of our patients—one with MPS II and one carrying a DSG2 cardiomyopathy allele—had structurally normal baseline echocardiograms, yet both belonged to disorders in which cardiac disease develops over time. Thus, a normal early study should support continued surveillance rather than reassurance alone. For disorders in which subclinical dysfunction precedes symptoms, such as dystrophinopathies, surveillance is increasingly based on scheduled imaging according to age and tissue-level assessment that can detect cardiac involvement before the ejection fraction declines [26]. This is illustrated by the neuromuscular patient in our cohort, whose left ventricular function declined between two annual studies. Conversely, gene-informed surveillance also helps avoid unnecessary screening. For example, longitudinal echocardiographic studies in hypermobile Ehlers–Danlos syndrome have not demonstrated an increased risk of aortic dilatation compared with the general population, and routine imaging is not recommended in the absence of other clinical findings [27].

A recurring challenge was determining whether a cardiac finding could be attributed to the identified genotype. Multilocus disease and dual molecular diagnoses are increasingly recognized with the widespread use of NGS, making it inappropriate to assume that every cardiovascular abnormality is explained by a single pathogenic variant [12,13]. We therefore adopted a conservative approach and excluded cases in which the cardiac finding could not reasonably be attributed to the established diagnosis. This approach strengthens the internal validity of the genotype-to-phenotype associations reported here, although the reported frequencies should be interpreted as conservative estimates.

It is also worth situating this kind of cohort within the wider landscape of genotype–phenotype resources. Our patients carry rare, high-penetrance variants in single genes, and the inferences we draw are made at the level of the individual case. Complementary resources operate at the population level: RAVAR curates published rare variant–trait associations at both gene and variant level [28], and GWAShug assembles the shared genetic architecture of complex traits from genome-wide association summary statistics [29]. Neither was used in the present analysis, and neither would be expected to capture Mendelian lesions of the kind described here. They do, however, indicate a direction of travel: linking deeply phenotyped Mendelian cases such as ours to population-scale rare-variant and complex-trait catalogues may eventually clarify why the cardiac expression of the same genotype varies so much between individuals, and which common-variant background modifies it.

Several limitations should be considered. This was a retrospective, single-center study based on patients referred for outsourced sequencing; therefore, referral and ascertainment bias are unavoidable, and the sample size was relatively small. Consequently, the reported frequencies should not be interpreted as population estimates. Sequencing was performed by external laboratories that did not provide raw sequencing data, preventing reanalysis of variants or further evaluation of variants of uncertain significance. As a result, a small number of diagnoses relied on such variants. Cardiac assessments were based on routine clinical echocardiography rather than a standardized research protocol, introducing some measurement variability, as illustrated by the discrepant M-mode ejection fraction measurements in one neuromuscular patient. Finally, long-term clinical outcomes were not systematically available. Larger, multicenter, prospectively phenotyped cohorts will be needed to validate these findings and establish disorder-specific cardiac surveillance recommendations.

Two further constraints follow from the points raised above. Because global longitudinal strain and, in most cases, cardiac magnetic resonance were not part of routine practice during the study period, early or subclinical myocardial involvement may have been missed, and the true frequency of functional abnormalities is likely to be underestimated. In addition, the echocardiograms were obtained at different ages, at different time points, and by different operators, so the cross-sectional proportions we report cannot describe the age-dependent evolution that characterises cardiac disease in the mucopolysaccharidoses, the dystrophinopathies, and the inherited cardiomyopathies; a prospective protocol with fixed intervals and standardised deformation imaging would be required for that. Finally, the assignment of each lesion as classic or incidental was made by a single investigator. Although the criteria were pre-specified and taken from OMIM and the disease-specific literature, the classification was not repeated independently, so residual observer bias cannot be excluded; the categories should be read as a descriptive framework rather than as adjudicated diagnoses.

## 4. Materials and Methods

### 4.1. Study Design and Setting

This retrospective, single-center study was conducted in the Division of Pediatric Genetics at a tertiary teaching hospital in Taipei, Taiwan. We reviewed patients for whom NGS had been requested through the division over an 8 yr period and who had also undergone cardiac assessment. The study was approved by the institutional review board (see the Institutional Review Board Statement), and all data were analyzed in de-identified form.

### 4.2. Case Ascertainment and Eligibility Criteria

Candidate patients were identified from the division’s outsourced testing log, which records every sample sent for external molecular testing together with the working clinical diagnosis. Patients were eligible if they had a molecularly confirmed genetic disorder and documented cardiovascular involvement—structural, functional, or arrhythmic—in the medical record. Patients were excluded if the molecular diagnosis remained unconfirmed (for example, when a suspected condition had been ruled out or was based only on a variant of uncertain significance without a supporting phenotype), if no cardiac assessment was available, or if the documented cardiac finding could not reasonably be attributed to the genetic diagnosis, most commonly because it represented a coincidental structural lesion unrelated to the causative gene. All exclusions and the reasons for exclusion were recorded.

**Figure 4:**
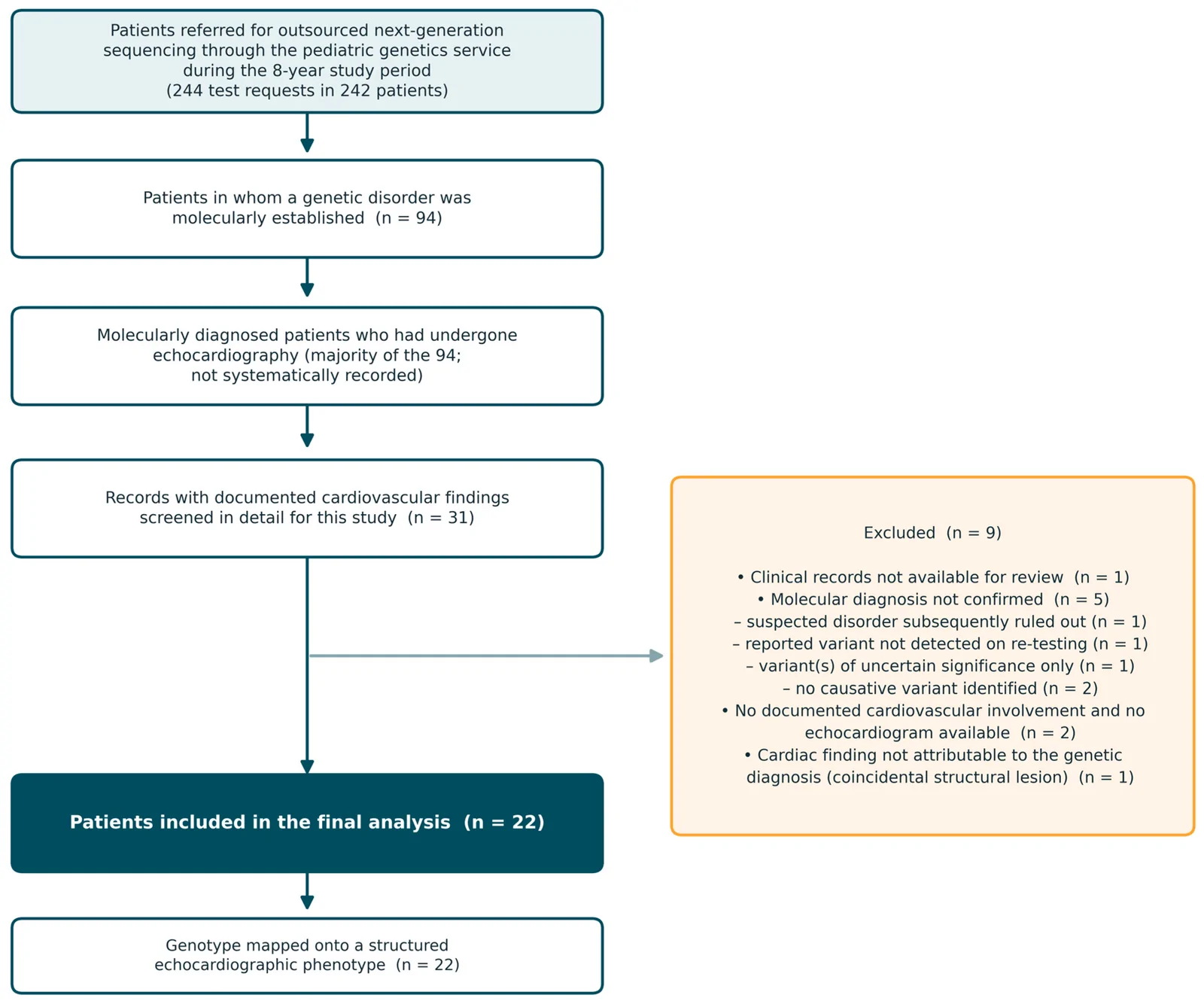
Study flow diagram for patient selection. Candidate records identified from the division’s outsourced-testing log were screened against the eligibility criteria described in this section; the number excluded at each step and the reason for every exclusion are shown. Records were screened from the divisional testing log before diagnostic confirmation was independently verified for this study, which is why an unconfirmed molecular diagnosis appears among the exclusion reasons.

Because cardiovascular involvement was defined at the level of the individual patient, two situations required an explicit rule, which was set before analysis. First, a patient whose echocardiogram was structurally normal at baseline was still eligible when the established genotype is one in which cardiac disease characteristically develops over time and the child was therefore enrolled in cardiac surveillance; two patients entered the cohort on this basis, and both are identified as such in Table 1 and in Section 2.2. Second, a patient whose cardiac finding could not reasonably be attributed to the established diagnosis was excluded even when the finding itself was unequivocal, as in the case of a coincidental structural lesion unrelated to the causative gene. Applying these criteria to the 31 candidate records identified 22 eligible patients and nine exclusions; the selection process and the reason for every exclusion are summarised in Figure 4.

### 4.3. Molecular Diagnosis

Molecular testing was performed by external commercial laboratories and included targeted gene panels, whole-exome sequencing, whole-genome sequencing, chromosomal microarray analysis, and multiplex ligation-dependent probe amplification, according to the referring clinician’s request. To preserve vendor anonymity, the laboratories are referred to only as Labs A–E. Because testing was outsourced, raw sequencing data were not available for reanalysis; therefore, the molecular diagnoses and reported variants were based on each laboratory’s clinical report. Sequence variants were classified as pathogenic, likely pathogenic, or variants of uncertain significance according to the joint consensus standards of the American College of Medical Genetics and Genomics and the Association for Molecular Pathology [30]. Gene symbols follow the HGNC nomenclature and are presented in italics.

### 4.4. Cardiovascular Phenotyping

Cardiac phenotypes were obtained from routine clinical transthoracic echocardiographic reports and supplemented, where available, by electrocardiography, cardiac magnetic resonance imaging, and cardiology records. For each patient, we recorded left ventricular ejection fraction, chamber dimensions, septal and posterior wall thickness, valve morphology with any regurgitation or stenosis, atrial and ventricular septal integrity, ductal patency, and great-vessel anatomy. Ejection fraction and fractional shortening were recorded as reported. Aortic and aortic-arch dimensions were expressed as body surface area-adjusted *z*-scores provided by the interpreting laboratory. The echocardiographic probability of pulmonary hypertension was assigned according to the 2022 ESC/ERS criteria, based on peak tricuspid regurgitation velocity together with supportive right-heart findings [31]. Each finding was classified into one of nine lesion categories: septal hypertrophy or hypertrophic cardiomyopathy, left ventricular systolic dysfunction or dilated cardiomyopathy, valvular regurgitation, valvular stenosis, septal defect or shunt, great-vessel or aortic abnormality, pulmonary hypertension, cardiac tumor, and arrhythmia or sudden death. Patients could contribute to more than one category. Each lesion was further classified as classic, indicating an expected manifestation of the underlying disorder, or incidental, based on established gene–disease relationships.

Several definitions were fixed in advance. Septal hypertrophy, and the descriptor “HCM phenotype”, were applied when the end-diastolic interventricular septal thickness exceeded the upper limit of the age- and body-surface-area-specific reference range quoted on the reporting laboratory’s own worksheet, with the pattern of hypertrophy and the left-ventricular outflow-tract gradient recorded where available; the term is descriptive of the echocardiographic appearance and is not intended to imply a diagnosis of sarcomeric hypertrophic cardiomyopathy. Valvular regurgitation and stenosis were graded as reported (trivial, mild, moderate, or severe). Aortic and arch dimensions were expressed as body-surface-area-adjusted z-scores as provided by the interpreting laboratory. Global longitudinal strain was not part of the routine protocol during most of the study period and was therefore not available for systematic analysis. The designation of each lesion as classic or incidental was made against established gene–disease relationships as catalogued in OMIM and in the disease-specific literature cited in the Discussion, rather than by impression, and the criteria were fixed before the records were reviewed. The classification was carried out by the first author, a pediatric geneticist, working from the original echocardiographic reports issued by the pediatric cardiologists who had performed the studies; it was not duplicated independently by a second assessor, and this is acknowledged as a limitation. The individual echocardiographic measurements underlying these classifications are provided in Table S1.

### 4.5. Data De-Identification and Management

All records were de-identified before analysis. Patient names, medical record numbers, and dates of birth were removed and replaced with sequential study identifiers (CV-001 onward). To allow investigators to re-link cases to the original records within the hospital’s secure system without exposing patient identifiers in the working dataset, each entry retained only internal source locators—the year, row, and case number from the original testing log. Identifiable information remained exclusively within the institutional system and was not included in the analytical dataset, figures, or tables.

### 4.6. Analysis and Presentation

Given the descriptive nature of the study and the small sample size, analyses were limited to counts and proportions, and no formal hypothesis testing was performed. Data were tabulated in a spreadsheet, and figures were generated using Python 3.12.3 with Matplotlib 3.10.8. The genotype-to-cardiac-phenotype matrix (Figure 2) was constructed by cross-tabulating each patient’s lesion categories against the corresponding causative gene or locus.

## 5. Conclusions

In this single-center series based on eight years of outsourced sequencing, the cardiovascular findings in children with syndromic and metabolic disorders generally reflected the underlying genotype. Some lesions were essentially disease-specific and, on their own, pointed to the diagnosis, whereas others—chiefly septal defects and atrioventricular valve regurgitation—recurred across unrelated genes. The practical implication is that, once a molecular diagnosis has been established, cardiac assessment should be guided by the genotype rather than by symptoms. Baseline and serial echocardiography are warranted even when the initial study is normal in patients with genotypes known to develop cardiac disease over time, while unnecessary screening should be avoided in conditions associated with minimal cardiac risk. Translating these patterns into disorder-specific surveillance strategies will require larger, prospectively phenotyped, multicenter cohorts. In the meantime, pairing every molecular diagnosis with a structured cardiac phenotype is a practical step that clinical genetics services can readily implement.

## Figures and Tables

**Table 1:** Cardiovascular involvement in 22 patients with syndromic and metabolic genetic disorders, grouped by disorder category.

Case	Sex	Genetic Diagnosis	Gene/Locus	LVEF (%)	Cardiovascular Phenotype (Echocardiography)	Lesion Class	Age at Genetic Dx/Last Echo (y)
**RASopathy (Noonan Spectrum; *n* = 2)**							
CV-017	F	Noonan-like disorder w/loose anagen hair 2	*PPP1CB*	73.7	Septal hypertrophy; small ASD/PFO	Classic	9.4/9.3
CV-025	F	Noonan-like syndrome	*SHOC2*	69.3	Pulmonary valve stenosis; mild MVP/MR	Classic	2.8/2.8
**Mucopolysaccharidoses (MPS; *n* = 4)**							
CV-007	F	MPS VII (Sly)	*GUSB*	60.0	Thick IVS; mitral + aortic valve thickening (MR/AR); small ASD	Classic	4.8/10.5
CV-015	M	MPS IVA (Morquio A)	*GALNS*	62.7	Mild septal thickening; trivial TR/MR	Classic	3/5.5
CV-019	M	MPS IIIC (Sanfilippo C)	*HGSNAT*	67.3	Mild aortic regurgitation	Classic	9/8.9
CV-020	M	MPS II (Hunter)	*IDS*	65.9	Normal baseline echo	—	0.5/1.6
**CHARGE syndrome (*n* = 3)**							
CV-001	F	CHARGE syndrome	*CHD7*	76.7	Mild septal thickening; otherwise, structurally normal	Incidental	17.5/21.8
CV-009	M	CHARGE syndrome	*CHD7*	76.6	PDA (ligated); ASD/PFO (spontaneously closed)	Classic	0/1.3
CV-011	F	CHARGE syndrome	*CHD7*	83.8	PDA (device-closed); dilated hypertrophic LV; est. PAP 45.6	Classic	10.9/10.9
**Connective-tissue disorder (*n* = 2)**							
CV-002	F	Ehlers–Danlos syndrome	*TNXB*	66.6	Small ASD/PFO; trivial MR	Classic	14.2/17.6
CV-016	F	Osteogenesis imperfecta + EDS	*COL1A1*	59.7	Secundum ASD; mild AR/MR/TR	Classic	29.8/31.8
**Primary/genetic cardiomyopathy & channelopathy (*n* = 3)**							
CV-024	M	Dilated cardiomyopathy (familial)	FLNC	30 (nadir 18)	DCM, LVEF 24–30%; s/p VSO + CRT-D; BAV; moderate MR	Classic	14.3/15.3
CV-029	F	Cardiomyopathy/channelopathy	*MYBPC3*	—	OHCA → brain death; suspected long QT/channelopathy	Classic	11.7/—
CV-026	M	Currarino syndrome + DCM risk allele	*MNX1/DSG2*	68.7	Echo normal (shunts closed); DSG2-based CM surveillance	—	0.1/0.4
**Neuromuscular disorder (*n* = 2)**							
CV-013	M	Duchenne muscular dystrophy	*DMD*	62.4	Mild septal hypertrophy; trivial-to-mild TR; preserved LVEF; ECG left-ventricular hypertrophy (cardiomyopathy surveillance)	Incidental	4/3.9
CV-014	M	Limb-girdle MD/EBS-MD	*PLEC*	31–53 *	Interval LV systolic dysfunction; abnormal septal motion	Classic	19.9/22.8
**Chromosomal/contiguous-gene syndrome (*n* = 3)**							
CV-022	F	22q11.2 deletion (DiGeorge)	*22q11.2 del*	60.2	VSD: spontaneously closed	Classic	13.2/13.3
CV-028	F	Tuberous sclerosis complex	*16p13.3 del (TSC2/PKD1)*	64.8	Multiple cardiac rhabdomyomas; small ASD/PFO	Classic	0.1/0
CV-030	M	Williams–Beuren syndrome	*7q11.23 del*	64.7	Supravalvular/peripheral PS; small ascending aorta (elastin arteriopathy)	Classic	0.1/0.2
**Other metabolic/syndromic (*n* = 3)**							
CV-010	M	Kabuki syndrome	*KMT2D*	68.2	PLSVC; BAV; aortic-arch anomaly; MVP/MR; mild MS	Classic	11.8/15.1
CV-012	F	Methylmalonic aciduria (mut0)	*MMUT*	56.3	ASD/PFO + small PDA; low-normal LVEF	Incidental	0/0
CV-027	M	Alagille syndrome	*JAG1*	63.6	Peripheral pulmonary stenosis; small ASD/PFO	Classic	0.3/0.2

* CV-014: on the 2025 study the ejection fraction differed between methods (M-mode 31%, Simpson biplane 53%); both values are reported. Abbreviations: AR, aortic regurgitation; ASD, atrial septal defect; BAV, bicuspid aortic valve; CRT-D, cardiac resynchronization therapy defibrillator; DCM, dilated cardiomyopathy; EBS-MD, epidermolysis bullosa simplex with muscular dystrophy; LVEF, left ventricular ejection fraction; MR, mitral regurgitation; MPS, mucopolysaccharidosis; MVP, mitral valve prolapse; OHCA, out-of-hospital cardiac arrest; PDA, patent ductus arteriosus; PFO, patent foramen ovale; PLSVC, persistent left superior vena cava; PS, pulmonary stenosis; TR, tricuspid regurgitation; VSD, ventricular septal defect; VSO, ventricular septal occlusion. “Lesion class” indicates whether the cardiovascular finding is a classic/expected manifestation of the underlying disorder or an incidental finding. CV-014: The 2024 echocardiogram was normal; however, interval left ventricular systolic dysfunction was identified on the 2025 study (M-mode ejection fraction, 31–47%; Simpson method, 53%). Biplane Simpson and global longitudinal strain measurements are recommended for definitive assessment. CV-029: No echocardiogram was available because the patient died following OHCA; the MYBPC3 variant was classified as a variant of uncertain significance.

**Table 2:** Frequency of cardiovascular manifestations (*n* = 22 patients; a patient may have more than one).

Cardiovascular Manifestation	Patients (*n*)	% of the Cohort
Septal defect/shunt	10	45%
Valvular regurgitation	9	41%
Septal hypertrophy/HCM phenotype	5	23%
Valvular stenosis	4	18%
Great-vessel/aortopathy	3	14%
LV systolic dysfunction/DCM	2	9%
Pulmonary hypertension	1	5%
Cardiac tumor	1	5%
Arrhythmia/sudden death	1	5%

**Table 3:** Molecular spectrum of the 22 patients: causative genes, variants, and affected functional pathways.

Case	Gene	Variant(s) (cDNA; Protein)	Zygosity/Inheritance	Variant Type	ACMG	Molecular Pathway
CV-001	*CHD7* (OMIM *608892)	c.2189C>T (p.Thr730Ile); c.8020G>T (p.Glu2674Ter)	comp. het	missense + nonsense	P/LP	Chromatin/transcriptional regulation
CV-009	*CHD7* (OMIM *608892)	c.914del (p.Asn305ThrfsTer14)	het (de novo)	frameshift	P	Chromatin/transcriptional regulation
CV-011	*CHD7* (OMIM *608892)	c.6104-2A>G; c.8189C>T (p.Ala2730Val)	comp. het	splice + missense	P/LP	Chromatin/transcriptional regulation
CV-010	*KMT2D* (OMIM *602113)	c.3906+2T>C	het	splice	P	Chromatin/transcriptional regulation
CV-007	*GUSB* (OMIM *611499)	c.104C>A (p.Ser35Ter); c.1454C>T (p.Ser485Phe)	comp. het	nonsense + missense	P/LP	Lysosomal GAG catabolism
CV-015	*GALNS* (OMIM *612222)	c.953T>G	hom/comp. het	missense	LP	Lysosomal GAG catabolism
CV-019	*HGSNAT* (OMIM *610453)	c.607C>T (p.Arg203Ter)	hom/comp. het	nonsense	P	Lysosomal GAG catabolism
CV-020	*IDS* (OMIM *300823)	c.1181-15C>A	hemizygous (de novo)	splice	LP	Lysosomal GAG catabolism
CV-017	*PPP1CB* (OMIM *600590)	c.548A>C (p.Glu183Ala)	het	missense	LP	RAS–MAPK signalling
CV-025	*SHOC2* (OMIM *602775)	c.4A>G (p.Ser2Gly)	het	missense	P	RAS–MAPK signalling
CV-029	*MYBPC3* (OMIM *600958)	c.104G>A (p.Arg35Gln)	het	missense	VUS	Sarcomere & cytoskeleton
CV-024	*FLNC* (OMIM *102565)	c.5647del (p.Val1883fs*70)	het	frameshift	P	Sarcomere & cytoskeleton
CV-013	*DMD* (OMIM *300377)	c.7354G>T (p.Glu2452Ter)	hemizygous	nonsense	P	Sarcomere & cytoskeleton
CV-014	*PLEC* (OMIM *601282)	c.9343C>T; c.13192G>A	comp. het	missense	LP	Sarcomere & cytoskeleton
CV-026	*DSG2* (OMIM *125671); *MNX1* (OMIM *142994)	DSG2 c.81+1G>A	het	splice	LP	Desmosomal adhesion
CV-016	*COL1A1* (OMIM *120150)	c.2550del (p.Gly851fs*257)	het	frameshift	P	Extracellular matrix (collagen/elastin)
CV-002	*TNXB* (OMIM *600985)	p.Asn1541Thr; p.Gly2922Ser	comp. het	missense	VUS/LP	Extracellular matrix (collagen/elastin)
CV-030	*ELN* (OMIM *130160); *7q11.23 del*	7q11.23 deletion incl. ELN	het (del)	contiguous-gene deletion	P	Extracellular matrix (collagen/elastin)
CV-027	*JAG1* (OMIM *601920)	c.2122_2125del (p.Gln708fs)	het (de novo)	frameshift	P	Notch signalling
CV-028	*TSC2* (OMIM *191092); *16p13.3 del*	16p13.3 deletion (TSC2–PKD1)	het (del)	contiguous-gene deletion	P	mTOR regulation
CV-022	*TBX1* (OMIM *602054); *22q11.2 del*	22q11.2 deletion	het (del)	contiguous-gene deletion	P	Conotruncal patterning (TBX1 region)
CV-012	*MMUT* (OMIM *609058)	c.1106G>A (p.Arg369His); c.1677-1G>A	comp. het	missense + splice	P/LP	Organic-acid metabolism

Abbreviations: ACMG, American College of Medical Genetics and Genomics; comp. het, compound heterozygous; LP, likely pathogenic; P, pathogenic; VUS, variant of uncertain significance. Variants are reported as provided by the referring laboratories; gene symbols follow HGNC nomenclature.

## Data Availability

The original contributions presented in this study are included in the article and Supplementary Materials. Further inquiries can be directed to the corresponding authors.

## Supplementary Materials

The following supporting information can be downloaded at https://www.mdpi.com/article/10.3390/ijms27167080/s1.

## References

[1] Yang Y., Muzny D.M., Reid J.G., Bainbridge M.N., Willis A., Ward P.A., Braxton A., Beuten J., Xia F., Niu Z. (2013). Clinical whole-exome sequencing for the diagnosis of Mendelian disorders. N. Engl. J. Med..

[2] Retterer K., Juusola J., Cho M.T., Vitazka P., Millan F., Gibellini F., Vertino-Bell A., Smaoui N., Neidich J., Monaghan K.G. (2016). Clinical application of whole-exome sequencing across clinical indications. Genet. Med..

[3] Pandey R., Brennan N.F., Trachana K., Katsandres S., Bodamer O., Belmont J., Veenstra D.L., Peng S. (2025). A meta-analysis of diagnostic yield and clinical utility of genome and exome sequencing in pediatric rare and undiagnosed genetic diseases. Genet. Med..

[4] Gorukmez O., Gorukmez O., Topak A. (2023). Clinical exome sequencing findings in 1589 patients. Am. J. Med. Genet. A.

[5] Ferreira C.R., Rahman S., Keller M., Zschocke J., ICIMD Advisory Group (2021). An international classification of inherited metabolic disorders (ICIMD). J. Inherit. Metab. Dis..

[6] Tarailo-Graovac M., Shyr C., Ross C.J., Horvath G.A., Salvarinova R., Ye X.C., Zhang L.H., Bhavsar A.P., Lee J.J., Drögemöller B.I. (2016). Exome sequencing and the management of neurometabolic disorders. N. Engl. J. Med..

[7] Hoytema van Konijnenburg E.M.M., Wortmann S.B., Koelewijn M.J., Tseng L.A., Houben R., Stöckler-Ipsiroglu S., Ferreira C.R., van Karnebeek C.D.M. (2021). Treatable inherited metabolic disorders causing intellectual disability: 2021 review and digital app. Orphanet J. Rare Dis..

[8] Borakay D., Karimova M., Noyan B., Akalin F., Elcioglu H.N. (2026). Cardiac involvement across mucopolysaccharidosis subtypes: Insights from a longitudinal single-center study. J. Pediatr. Endocrinol. Metab..

[9] Mekdade T., Bérat C.M., Schiff M., Gaschignard M., Bouchereau J., Arnoux J.B., Francoz C., Servais A., Dao M., Lacroix V. (2026). Long term follow-up after transplantation in propionic acidemia: A retrospective French pediatric and adult cohort study. J. Inherit. Metab. Dis..

[10] Orlova A., Shatokhina O., Guseva D., Demina N., Polyakov A., Ryzkova O. (2026). Genetic spectrum of non-PTPN11 variants in Noonan Syndrome and related RASopathies: Findings from a Russian cohort. Clin. Genet..

[11] Peretto G., Merlo M., Ambrosi A., Bacigalupi E., Villatore A., Molinari L., Anguera I., Claver E., Dal Ferro M., Suwalski P. (2026). Major arrhythmias in non-dilated left ventricular cardiomyopathy: A novel prediction score. Eur. Heart J..

[12] Posey J.E., Harel T., Liu P., Rosenfeld J.A., James R.A., Coban Akdemir Z.H., Walkiewicz M., Bi W., Xiao R., Ding Y. (2017). Resolution of disease phenotypes resulting from multilocus genomic variation. N. Engl. J. Med..

[13] Mitani T., Isikay S., Gezdirici A., Gulec E.Y., Punetha J., Fatih J.M., Herman I., Akay G., Du H., Calame D.G. (2021). High prevalence of multilocus pathogenic variation in neurodevelopmental disorders in the Turkish population. Am. J. Hum. Genet..

[14] Yang Y., Muzny D.M., Xia F., Niu Z., Person R., Ding Y., Ward P., Braxton A., Wang M., Buhay C. (2014). Molecular findings among patients referred for clinical whole-exome sequencing. JAMA.

[15] Xing G., Chen L., Lv L., Hu C., Liu S., Duan Y., Li J., Wang Q., Li X. (2025). Genetic and clinical characterization of FLNC variants in Chinese patients with cardiomyopathy. J. Cardiovasc. Dev. Dis..

[16] Ertaş K., Gül Ö., Bozacı A.E., Bilgin H. (2025). Evaluation of cardiac function in pediatric patients diagnosed with mucopolysaccharidosis (MPS) and use of annular plane systolic excursion (APSE) to evaluate systolic function. Mol. Genet. Metab..

[17] Hamers J., Sen P., Murthi S.R., Papanakli L., von Stumm M., Baessato F., Cleuziou J., Meierhofer C., Ewert P., Dendorfer A. (2025). Trametinib alters contractility of paediatric Noonan syndrome-associated hypertrophic myocardial tissue slices. ESC Heart Fail..

[18] Millette T.J., McCulloch M.A., Yao Q., Hainstock M.R. (2025). RVOT stenting and trametinib in an infant with Noonan syndrome, pulmonary stenosis, and hypertrophic cardiomyopathy. JACC Case Rep..

[19] Brodehl A., Ferrier R.A., Hamilton S.J., Greenway S.C., Brundler M.-A., Yu W., Gibson W.T., McKinnon M.L., McGillivray B., Alvarez N. (2016). Mutations in *FLNC* are associated with familial restrictive cardiomyopathy. Hum. Mutat..

[20] Brodehl A., Meshkov A., Myasnikov R., Kiseleva A., Kulikova O., Klauke B., Sotnikova E., Stanasiuk C., Divashuk M., Pohl G.M. (2021). Hemi- and homozygous loss-of-function mutations in *DSG2* (desmoglein-2) cause recessive arrhythmogenic cardiomyopathy with an early onset. Int. J. Mol. Sci..

[21] Kiener A., Lantin M.R.L., Lawrence E.J., Morris S.A., Sheth S.S. (2024). Fetal diagnosis of supravalvular aortic stenosis and pulmonary stenosis in a family with non-syndromic elastin mutation. Pediatr. Cardiol..

[22] Zhao Y., Wang Y., Shi L., McDonald-McGinn D.M., Crowley T.B., McGinn D.E., Tran O.T., Miller D., Lin J.R., Zackai E. (2023). Chromatin regulators in the TBX1 network confer risk for conotruncal heart defects in 22q11.2DS. npj Genom. Med..

[23] Ogawa Y., Yamamoto A., Yamazawa S., Ikemura M., Hirata Y., Inuzuka R. (2025). Decreased smooth muscle cells and fibrous thickening of the tunica media in peripheral pulmonary artery stenosis in Alagille syndrome. Cardiovasc. Pathol..

[24] Baba S., Ozawa J., Tsuru H., Nukaga S., Tsukada M., Nirei J., Abe T., Numano F., Saitoh A. (2026). Successful everolimus therapy for atrioventricular block due to cardiac rhabdomyoma associated with tuberous sclerosis complex. JACC Case Rep..

[25] Belur L.R., Huber A.K., Mantone H., Robertson M., Smith M.C., Karlen A.D., Kitto K.F., Ou L., Whitley C.B., Braunlin E. (2024). Intrathecal or intravenous AAV9-IDUA/RGX-111 at minimal effective dose prevents cardiac, skeletal and neurologic manifestations of murine MPS I. Mol. Ther. Methods Clin. Dev..

[26] McDiarmid A., Augustine D., Coats C., Myerson S.G., Hughes M., Villa C., Soslow J., Kadhim K., Kerr A., Kuhwald L. (2026). Cardiac MRI in cardiac dystrophinopathy: Recommendations on imaging. Open Heart.

[27] Lahey H., Shin H., Myers K., McBride K.L. (2024). Longitudinal echocardiography in pediatric patients with hypermobile Ehlers-Danlos Syndrome. Am. J. Med. Genet. A.

[28] Cao C., Shao M., Zuo C., Kwok D., Liu L., Ge Y., Zhang Z., Cui F., Chen M., Fan R. (2024). RAVAR: A curated repository for rare variant–trait associations. Nucleic Acids Res..

[29] Cao C., Tian M., Li Z., Zhu W., Huang P., Yang S. (2025). GWAShug: A comprehensive platform for decoding the shared genetic basis between complex traits based on summary statistics. Nucleic Acids Res..

[30] Richards S., Aziz N., Bale S., Bick D., Das S., Gastier-Foster J., Grody W.W., Hegde M., Lyon E., Spector E. (2015). Standards and guidelines for the interpretation of sequence variants: A joint consensus recommendation of the American College of Medical Genetics and Genomics and the Association for Molecular Pathology. Genet. Med..

[31] Humbert M., Kovacs G., Hoeper M.M., Badagliacca R., Berger R.M.F., Brida M., Carlsen J., Coats A.J.S., Escribano-Subias P., Ferrari P. (2022). 2022 ESC/ERS Guidelines for the diagnosis and treatment of pulmonary hypertension. Eur. Heart J..

