# Supplementary material for: From Genotype to Cardiac Phenotype: Cardiovascular Involvement in Syndromic and Metabolic Disorders
Source: Int J Mol Sci. 2026 Aug 7;27(16):7080. doi: 10.3390/ijms27167080 (PMC13513472; doi:10.3390/ijms27167080)
Supplement: Supplementary file 1 [file ijms-27-07080-s001.zip › ijms-4459257-supplementary.pdf]

Table S1. Age, cardiac follow-up, and quantitative echocardiographic parameters of the 22 patients.

Study_ID	Diagnosis	Age at genetic diagnosis (y)	No. of echocardiograms	Age at first echo (y)	Age at last echo (y)	Cardiac follow-up (y)	LVEF (%)	IVSd (cm)	LVPWd (cm)	LVIDd (cm)	LVIDs (cm)	TR peak gradient (mmHg)	Index echo conclusion
CV-001	CHARGE syndrome	17.5	2	17.2	21.8	4.6	76.7	0.929 (H) [ref 0.48–0.84]	0.715 [ref 0.42–0.94]	3.470 (L) [ref 3.57–4.87]	1.930 (L) [ref 2.15–3.27]		WNL
CV-002	Ehlers-Danlos syndrome	14.2	3	8.1	17.6	9.6	66.6	0.662 [ref 0.53–0.98]	0.662 [ref 0.50–1.07]	4.500 [ref 3.84–5.26]	2.850 [ref 2.28–3.51]		ASD/PFO MR
CV-007	MPS VII (Sly)	4.8	23	0.6	10.5	9.9	60	0.875 (H) [ref 0.38–0.74]	0.621 [ref 0.37–0.81]	3.851 [ref 3.11–4.36]	2.641 [ref 1.81–2.94]		ASDII Thick IVS MR AR
CV-009	CHARGE syndrome	0	2	0	1.3	1.3	76.6	0.529 [ref 0.25–0.54]	0.504 [ref 0.28–0.54]	2.670 [ref 1.94–2.80]	1.510 [ref 1.10–1.91]		PDA LIG ASD/PFO SPC
CV-010	Kabuki syndrome	11.8	23	0	15.1	15.1	68.2	0.660 [ref 0.47–0.83]	0.770 [ref 0.41–0.93]	4.560 [ref 3.56–4.82]	2.830 [ref 2.15–3.27]		MS PLSVC MVP MR BAV COA? ARSA
CV-011	CHARGE syndrome	10.9	6	10.7	10.9	0.2	83.8	0.586 (H) [ref 0.25–0.53]	0.663 (H) [ref 0.28–0.54]	2.750 (H) [ref 1.90–2.72]	1.350 [ref 1.08–1.88]		PDA ADOI Hypertrophic LV
CV-012	Methylmalonic aciduria	0	1	0	0	0	56.3						ASD/PFO PDA
CV-013	Duchenne muscular dystrophy	4	1	3.9	3.9	0	62.4	0.756 (H) [ref 0.34–0.64]	0.618 [ref 0.35–0.67]	3.730 [ref 2.73–3.99]	2.500 [ref 1.59–2.59]		TR
CV-014	Limb-girdle MD / EBS-MD	19.9	4	17.4	22.8	5.5	34.1	0.794 [ref 0.61–1.10]	0.832 [ref 0.51–1.11]	5.440 [ref 4.06–5.77]	4.540 (H) [ref 2.44–3.93]		
CV-015	MPS IVA (Morquio A)	3	13	0.1	5.5	5.4	62.7	0.721 (H) [ref 0.33–0.63]	0.559 [ref 0.33–0.63]	3.135 [ref 2.59–3.70]	2.108 [ref 1.53–2.42]		TR MR
CV-016	Osteogenesis imperfecta + EDS	29.8	2	29.4	31.8	2.4	59.7	0.820 [ref 0.70–1.10]	0.820 (L) [ref 0.90–1.10]	4.500 [ref 3.70–5.30]	3.100 [ref 2.40–3.60]		Normal sizes of cardiac chambers ; ASD, secudum type, left to right shunt wth maxial diameter: 0.34cm, Qp/Qs =1.0 ; Preserved global contractility of left ventr
CV-017	Noonan-like disorder	9.4	2	6.8	9.3	2.5	73.7	0.807 (H) [ref 0.37–0.70]	0.731 [ref 0.36–0.79]	3.230 [ref 3.00–4.24]	1.890 [ref 1.78–2.82]		Thick IVS ASD/PFO
CV-019	MPS IIIC	9	1	8.9	8.9	0	67.3	0.715 [ref 0.48–0.84]	0.715 [ref 0.42–0.94]	4.320 [ref 3.57–4.86]	2.720 [ref 2.15–3.27]		AR MR
CV-020	MPS II (Hunter)	0.5	5	0.2	1.6	1.5	65.9	0.468 [ref 0.27–0.59]	0.432 [ref 0.31–0.61]	3.225 [ref 2.34–3.46]	2.090 [ref 1.40–2.20]		WNL
CV-022	22q11.2 deletion	13.2	5	0.9	13.3	12.4	60.2	0.756 [ref 0.49–0.85]	0.756 [ref 0.43–0.95]	4.390 [ref 3.70–4.92]	2.990 [ref 2.19–3.31]		VSD SPC
CV-024	Dilated cardiomyopathy (FLNC)	14.3	39	0.3	15.3	15	30.2	1.120 [ref 0.64–1.13]	1.170 (H) [ref 0.51–1.11]	8.170 (H) [ref 4.23–5.90]	6.960 (H) [ref 2.48–4.07]		VSD VSO BAV AS AR DCM decreased LVEF s/p CRTD
CV-025	Noonan-like syndrome	2.8	3	0.5	2.8	2.3	69.3	0.623 (H) [ref 0.27–0.59]	0.467 [ref 0.31–0.61]	3.370 (H) [ref 2.28–3.36]	2.090 [ref 1.36–2.16]		PS MVP MR
CV-026	Currarino + DSG2	0.1	2	0	0.4	0.4	68.7	0.585 (H) [ref 0.25–0.53]	0.343 [ref 0.28–0.54]	2.380 [ref 1.90–2.72]	1.510 [ref 1.08–1.88]		PDA SPC PFO SPC
CV-027	Alagille syndrome	0.3	1	0.2	0.2	0	63.6	0.496 (H) [ref 0.23–0.49]	0.241 [ref 0.24–0.45]	1.810 [ref 1.51–2.13]	1.230 [ref 0.92–1.42]		ASD/PFO PPS
CV-028	Tuberous sclerosis	0.1	1	0	0	0	64.8	0.337 [ref 0.24–0.52]	0.297 [ref 0.26–0.48]	2.140 [ref 1.61–2.29]	1.430 [ref 0.99–1.48]		Nodules ASD/PFO
CV-029	Cardiomyopathy/channelopathy	11.7	0										No echocardiogram available (patient died following out-of-hospital cardiac arrest)
CV-030	Williams-Beuren	0.1	2	0	0.2	0.2	64.7						ASD/PFO SPC PDA SPC PPS DsAo

Notes: Values are M-mode measurements with the age- and body-surface-area-specific reference range quoted by the reporting laboratory; (H) and (L) denote a value above or below that range. Cardiac follow-up is the interval between the first and the last available echocardiogram. Global longitudinal strain was not part of the routine protocol during the study period and is therefore not available. Patient CV-029 died following an out-of-hospital cardiac arrest and no echocardiogram was available.
